# Different impacts of metabolic profiles on future risk of cardiovascular disease between diabetes with and without established cardiovascular disease: the Japan diabetes complication and its prevention prospective study 7 (JDCP study 7)

**DOI:** 10.1007/s00592-021-01773-z

**Published:** 2021-08-30

**Authors:** Mitsuyoshi Takahara, Naoto Katakami, Yasuaki Hayashino, Rimei Nishimura, Hiroaki Suzuki, Hitoshi Shimano, Narihito Yoshioka, Naoko Tajima, Yoshimitsu Yamasaki

**Affiliations:** 1 Department of Diabetes Care Medicine, Osaka University Graduate School of Medicine, 2-2 Yamadaoka, Suita City, Osaka 565-0871 Japan; 2 Department of Metabolic Medicine, Osaka University Graduate School of Medicine, 2-2 Yamadaoka, Suita City, Osaka 565-0871 Japan; 3 Department of Endocrinology, Tenri Hospital, 200 Mishimacho, Tenri City, Nara 632-8552 Japan; 4 Department of Diabetes, Metabolism and Endocrinology, Jikei University School of Medicine, 3-25-8 Nishi-Shimbashi, Minato-ku, Tokyo, 105-8461 Japan; 5 Department of Endocrinology and Metabolism, Faculty of Medicine, University of Tsukuba, 1-1-1 Tennodai, Tsukuba City, Ibaraki 305-8575 Japan; 6 NTT-East Sapporo Hospital, Minami 1 Jyo Nishi 15 Chome Chuo-Ku, Sapporo City, Hokkaido 060-0061 Japan; 7 Otemachi Place Medical Clinic, 2-3-1, Otemachi, Chiyoda-ku, Tokyo, 100-0004 Japan; 8 Nishi-Umeda Clinic, 3-3-45 Umeda, Kita-ku, Osaka City, Osaka 530-0001 Japan

**Keywords:** Future risk of cardiovascular disease, History of cardiovascular disease, Metabolic profile, Interaction effect

## Abstract

**Aims:**

Most risk calculators that predict future cardiovascular disease (CVD) by baseline profiles are originally developed for primary prevention, but some studies applied the calculators to secondary prevention. We compared the impact of baseline profiles on the future CVD risk between patients with diabetes with and without a CVD history.

**Methods:**

We analyzed a multicenter prospective cohort of 6338 Japanese patients with diabetes aged 40–74 years, including those with (*n* = 634) and without a CVD history (*n* = 5704). The future risk of CVD was investigated using the competing risk model, with adjustment for non-cardiovascular mortality.

**Results:**

During the median follow-up of 6.9 years, 413 CVD events were observed. The 8-year cumulative incidence rates of CVD were 21.5% and 7.2% in patients with and without a CVD history, respectively. A higher systolic blood pressure and lower high-density lipoprotein cholesterol levels were independently associated with a future CVD risk in patients without a CVD history (both *P* < 0.05), whereas they were not associated in those with a CVD history. The *P* values for interaction were 0.040 and 0.005, respectively. The male sex, an older age, a longer duration of diabetes, higher hemoglobin A1c levels, and higher low-density lipoprotein cholesterol levels were common independent risk factors regardless of CVD history (all *P* < 0.05).

**Conclusions:**

The prognostic impact of metabolic profiles on CVD risk would not be identical between patients with and without a CVD history, suggesting that it might be inappropriate to apply CVD risk calculators developed for primary prevention to patients with a CVD history.

**Supplementary Information:**

The online version contains supplementary material available at 10.1007/s00592-021-01773-z.

## Introduction

Cardiovascular disease (CVD) is a major cause of morbidity and mortality and is an enormous health care and economic burden [1–3]. A population with diabetes has a 2–4 times higher CVD risk than a population without diabetes [4]. The CVD risk will be increased in patients with poorly controlled metabolic profiles, including glucose, lipids, and blood pressure [5, 6], whereas the CVD risk can be modified by improving these metabolic profiles [7]. To date, several risk prediction models for future CVD incidence have been developed [5, 6]. They are often based on metabolic profiles and do not require detailed information of medication use and lifestyle interventions. They enable to convert the current control of metabolic profiles into the estimated absolute risk for future CVD incidence. The risk prediction models have been also used in many clinical studies to convert the improvement of metabolic control by an intervention into the estimated absolute risk reduction of future CVD incidence, regardless of patients’ CVD history [8–10]. However, these risk prediction models were originally developed from a population without a history of CVD [5, 6]. It remains unclear whether applying these risk prediction models to patients with a history of CVD would overestimate or underestimate the CVD risk reduction that corresponds to an improvement of metabolic profiles. This study aimed to compare the prognostic impact of metabolic profiles on the future CVD risk between patients with diabetes with and without a history of CVD.

## Materials and methods

### Study population

We analyzed a clinical database obtained from the Japan Diabetes Complication and its Prevention prospective study (JDCP study), a prospective, multicenter, cohort study that registered patients with diabetes in Japan [11–17]. In brief, the Japan Diabetes Society (JDS) conducted this study at 464 medical facilities specializing in diabetes, including university hospitals, local base hospitals, and clinics across Japan. The inclusion criteria were (i) patients with type 1 and 2 diabetes and (ii) those aged 40–74 years. The exclusion criteria were (i) unable to visit their outpatient clinic regularly, (ii) proliferative retinopathy, (iii) currently on dialysis, (iv) diagnosed with malignancy in the past five years, and (v) judged by the study investigator to be ineligible for study entry. The study subjects were recruited at medical facilities specializing in diabetes, and 7700 eligible patients who gave written informed consent were provisionally enrolled between June 2007 and November 2009. After excluding those who did not meet the study eligibility criteria, the JDCP study finally registered 6338 patients (Supplementary Figure S1). Patient information, including baseline characteristics and the occurrence of CVD events, was collected with the use of case report form, which was filled at baseline, and thereafter once every year.

### Ethics

The Declaration of Helsinki and the domestic ethical guideline applicable during the study period [18] were followed in the JDCP study. All participants provided written informed consent to participate in the registry. The JDCP study was approved by the JDS Ethics Review Committee for Scientific Surveys and Studies and the Ethics Committee of each participating institution (or an ad hoc ethics committee convened at the request of the principal investigator if the required review process could not be put in place at any of the participating institutions) and registered with the University Hospital Medical Information Network Center (UMIN) with the identifier UMIN000016519.

### Definitions

The occurrence of CVD was defined as a composite of (i) coronary artery disease (including myocardial infarction, angina pectoris, and coronary revascularization), (ii) hospital admission for heart failure, (iii) cerebrovascular disease (including stroke, transient ischemic attack, and cephalic revascularization), and (iv) peripheral artery disease (including peripheral artery disease, lower extremity amputation [without peripheral neuropathy], and peripheral revascularization). When CVD events were clinically diagnosed at individual centers, investigators reported the events, simultaneously submitting their supporting materials, including physiological and imaging tests. A review board of the working group on macrovascular disease finally confirmed the occurrence of the events, based on the submitted information. Angina pectoris was determined by the review board using the following criteria: ST-segment depression in load electrocardiogram, perfusion redistribution following defects in stress myocardial perfusion scintigraphy, or ≥ 75% stenosis in coronary angiography or multidetector computed tomography, whereas peripheral artery disease was determined using the following criteria: ≤ 0.9 of ankle brachial index, or ≥ 75% stenosis detected by angiography, vascular ultrasound, computed tomography, or magnetic resonance angiography. A history of CVD was based on medical records and medical interviews and diagnoses by attending physicians. The information about death and causes of death was obtained by attending physicians and was confirmed by the review board of the working group, based on copies of medical records and death certificates, whenever possible. Smoking history included both current and past smoking. Glycated hemoglobin (HbA1c) levels were expressed in accordance with the National Glycohemoglobin Standardization Program as recommended by the Japanese Diabetes Society [19].

### Statistical analyses

Data are presented as medians and interquartile ranges for continuous variables or as percentages for discrete variables, if not otherwise mentioned. A *P* value < 0.05 was considered statistically significant and 95% confidence intervals were reported where appropriate. Baseline characteristics were compared between patients with and without a history of CVD using the Mann–Whitney’s *U* test for continuous variables and the chi-squared test for discrete variables. The crude cumulative incidence of CVD was estimated using the cumulative incidence function, treating non-cardiovascular death as a competing risk. The association between baseline characteristics and CVD incidence was analyzed using Fine and Gray’s proportional hazards regression model for the subdistribution of competing risks, with adjustment for anti-diabetic, anti-hypertensive, and anti-hyperlipidemic medications as the stratification variables. The influence of a CVD history on the association between a baseline characteristic and future CVD risk was evaluated as an interaction effect, by entering the variable of the CVD history, that of the baseline characteristic, and their interaction term (i.e., the product of the two variables) together in the regression model. The significance of the interaction effect was judged by the fact that the regression coefficient for the interaction term was not zero. Missing data were addressed by multiple imputation using the chained equations method. In the procedure, we generated five imputed datasets and combined the analytic results based on Rubin’s rule. All statistical analyses were performed using R version 3.6.0 (R Development Core Team, Vienna, Austria).

## Results

Baseline characteristics are presented in Table 1. The median patient age and duration of diabetes were 62 (56–67) years and 9 (5–15) years, respectively, and 59.2% were men. Median HbA1c levels were 7.2% (6.7–7.9%) (55 [50–63] mmol/mol). Type 1 diabetes accounted for 6.2% of the study population. A total of 634 (10.0%) patients had a CVD history. Compared with patients without a CVD history, those with a CVD history had an older age, a longer duration of diabetes, a lower prevalence of type 1 diabetes, and a higher prevalence of male sex, insulin use, anti-hypertensive medication, and anti-hyperlipidemic medication. Patients with a CVD history had lower high-density lipoprotein (HDL) cholesterol levels, lower low-density lipoprotein (LDL) cholesterol levels, and higher triglycerides levels, whereas systolic blood pressure and HbA1c levels were similar in both patients.

**Table 1:** Baseline characteristics of the study population

	Overall	CVD history [–]	CVD history [ +]	*P* value
	(*n* = 6338)	(*n* = 5704)	(*n* = 634)	
Male sex	3749 (59.2%)	3297 (57.8%)	452 (71.3%)	< 0.001
Age (years)	62 (56–67)	61 (56–67)	65 (60–70)	< 0.001
Type of diabetes				
Type 1 diabetes	394 (6.2%)	383 (6.7%)	11 (1.7%)	< 0.001
Type 2 diabetes	5944 (93.8%)	5321 (93.3%)	623 (98.3%)	
No anti-diabetic medication	618 (10.4%)	574 (10.8%)	44 (7.1%)	< 0.001
Oral anti-diabetic medication	3680 (62.1%)	3331 (62.8%)	349 (56.4%)	
Insulin use	1629 (27.5%)	1403 (26.4%)	226 (36.5%)	
Duration of diabetes (years)	9 (5–15)	9 (5–15)	12 (5–18)	< 0.001
Smoking history	2392 (37.9%)	2143 (37.7%)	249 (39.3%)	0.47
Body mass index (kg/m^2^)	23.9 (21.7–26.4)	23.9 (21.7–26.5)	24.0 (22.0–26.3)	0.39
Systolic blood pressure (mmHg)	130 (120–138)	130 (120–138)	130 (120–140)	0.23
HbA1c (%)	7.2 (6.7—7.9)	7.2 (6.7—7.9)	7.2 (6.7—8.0)	0.18
(mmol/mol)	55 (50–63)	55 (50–63)	55 (50–64)	
LDL cholesterol (mg/dl)	111 (94–130)	112 (94–130)	104 (87–124)	< 0.001
HDL cholesterol (mg/dl)	56 (46–68)	56 (47–68)	52 (43–63)	< 0.001
Triglycerides (mg/dl)	104 (75—149)	103 (74—147)	117 (88—160)	< 0.001
Anti-hypertensive medication	2938 (46.5%)	2475 (43.5%)	463 (73.5%)	< 0.001
Anti-hyperlipidemic medication	2599 (41.1%)	2253 (39.6%)	346 (54.9%)	< 0.001

Data are presented as median (interquartile range), or number (percentage). Data were missing on anti-diabetic medication in 17 patients (0.3%), on duration of diabetes in 86 (1.4%), on smoking history in 21 (0.3%), on body mass index in 83 (1.3%), on systolic blood pressure in 79 (1.2%), on HbA1c in 22 (0.3%), on LDL cholesterol in 270 (4.3%), on HDL cholesterol in 109 (1.7%), on triglycerides in 3322 (52.4%), on anti-hypertensive medication in 14 (0.2%), and anti-hyperlipidemic medication in 17 (0.3%). CVD, cardiovascular disease; HDL, high-density lipoprotein; LDL, low-density lipoprotein

**Fig. 1:**
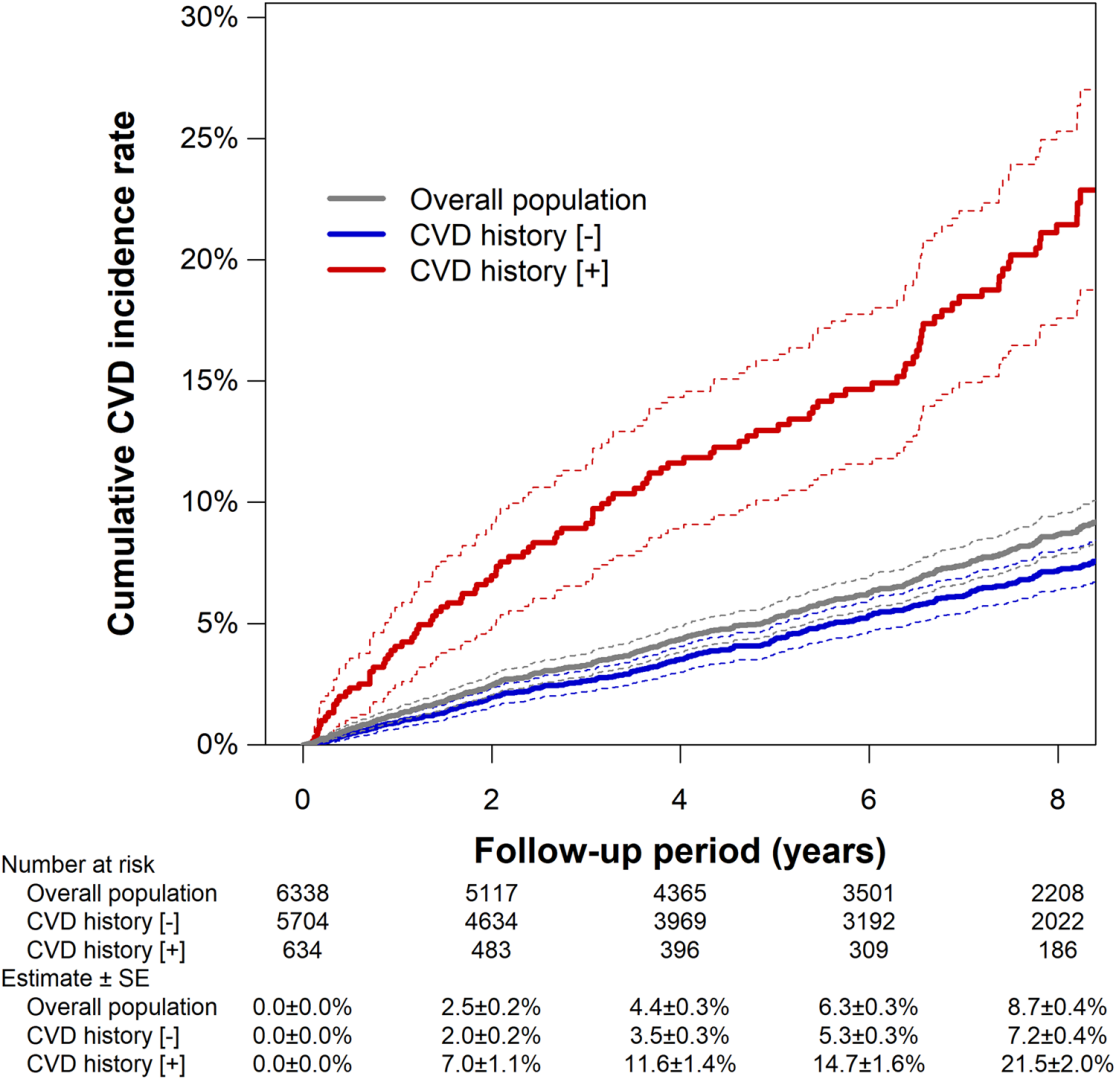
Cumulative incidence of CVD. The incidence rate was estimated by the cumulative incidence function in which non-cardiovascular death was treated as the competing risk. Dotted lines indicate 95% confidence intervals. SE, standard error

During the median follow-up of 6.9 (3.0–8.2) years, 413 patients experienced CVD events, whereas 149 patients died without experiencing CVD. Details of incident CVD are summarized in Supplementary Tables S1 and S2. The 8-year cumulative incidence of CVD (95% confidence interval) was estimated to be 7.2% (6.4–8.0%) in patients without a CVD history, whereas it was 21.5% (17.6–25.3%) in those with a CVD history (Fig. 1).

Crude interaction analysis revealed that systolic blood pressure and HDL cholesterol levels had a significantly different unadjusted hazard ratio for future CVD risk between patients with and without a CVD history (both *P* for interaction < 0.05) (Table 2). We subsequently performed multivariate analysis, in which the prognostic impact of these two variables was treated separately according to the CVD history, whereas the other baseline characteristics were regarded as having a shared prognostic impact. Consequently, as shown in Table 3, a higher systolic blood pressure and lower HDL cholesterol levels were significantly associated with future CVD risk in patients without a CVD history (both *P* < 0.05), whereas they were not associated in those with a CVD history; the p values for interaction were 0.040 and 0.005, respectively. The male sex, an older age, a longer duration of diabetes, lower body mass index, higher HbA1c levels, and higher LDL cholesterol levels were identified as common risk factors in patients with and without a CVD history (all *P* < 0.05). Supplementary Table S3 shows the crude association between baseline characteristics and the future risk of each CVD in patients with and without a CVD history.

**Table 2:** Impact of baseline characteristics on future CVD risk in patients with versus without CVD history

	Overall population	CVD history [–]	CVD history [ +]	*P* for interaction
Male sex	1.87 [1.49, 2.34]	2.03 [1.57, 2.62]	1.39 [0.88, 2.20]	0.16
Age (per 10 years)	1.46 [1.27, 1.68]	1.55 [1.33, 1.82]	1.14 [0.84, 1.56]	0.082
Type 1 diabetes	0.47 [0.25, 0.89]	0.42 [0.21, 0.84]	0.98 [0.24, 4.00]	0.29
Duration of diabetes (per 10 years)	1.25 [1.12, 1.40]	1.30 [1.15, 1.48]	1.11 [0.89, 1.39]	0.22
Smoking history	1.04 [0.86, 1.27]	1.08 [0.86, 1.36]	0.94 [0.63, 1.38]	0.53
Body mass index (per 5 kg/m^2^)	0.90 [0.79, 1.04]	0.94 [0.81, 1.09]	0.77 [0.56, 1.04]	0.23
Systolic blood pressure (per 10 mmHg)	1.09 [1.02, 1.16]	1.14 [1.06, 1.22]	0.96 [0.84, 1.09]	0.021
HbA1c (per 1% or per 10.9 mmol/mol)	1.13 [1.05, 1.22]	1.15 [1.07, 1.25]	1.06 [0.92, 1.24]	0.34
LDL cholesterol (per 20 mg/dl)	1.08 [1.01, 1.16]	1.08 [0.99, 1.17]	1.10 [0.97, 1.25]	0.79
HDL cholesterol (per 10 mg/dl)	0.79 [0.73, 0.84]	0.74 [0.68, 0.81]	0.92 [0.81, 1.05]	0.006
Triglycerides (per doubling)	1.21 [1.07, 1.37]	1.25 [1.08, 1.44]	1.11 [0.87, 1.42]	0.43

Data are presented as hazard ratios (HRs) for future CVD risk and their 95% confidence intervals, derived from the Fine and Gray’s proportional hazards regression model for the subdistribution of competing risks in which each variable of interest was entered as the explanatory variable, and anti-diabetic, anti-hypertensive, and anti-hyperlipidemic medications were entered as the stratification variables. Hazard ratios in the overall population were adjusted for CVD history. CVD, cardiovascular disease; HDL, high-density lipoprotein; LDL, low-density lipoprotein

**Table 3:** Impact of baseline characteristics on future CVD risk

	Adjusted hazard ratio	*P* for interaction
Male sex	1.77 [1.40, 2.24] (*P*<0.001)	
Age (per 10 years)	1.46 [1.25, 1.70] (*P*<0.001)	
Type 1 diabetes	0.78 [0.41, 1.47] (*P*=0.44)	
Duration of diabetes (per 10 years)	1.17 [1.03, 1.31] (*P*=0.012)	
Smoking history	1.04 [0.85, 1.27] (*P*=0.69)	
Body mass index (per 5 kg/m^2^)	0.86 [0.73, 1.01] (*P*=0.059)	
Systolic blood pressure (per 10 mmHg)		0.040
CVD history [–]	1.13 [1.05, 1.22] (*P*=0.001)	
CVD history [+]	0.97 [0.86, 1.10] (*P*=0.66)	
HbA1c (per 1% or per 10.9 mmol/mol)	1.13 [1.05, 1.22] (*P*=0.001)	
LDL cholesterol (per 20 mg/dl)	1.10 [1.02, 1.18] (*P*=0.009)	
HDL cholesterol (per 10 mg/dl)		0.005
CVD history [–]	0.77 [0.70, 0.84] (*P*<0.001)	
CVD history [+]	0.96 [0.84, 1.10] (*P*=0.53)	
Triglycerides (per doubling)	1.03 [0.89, 1.20] (*P*=0.69)	
CVD history	2.61 [2.03, 3.37] (*P*<0.001)	

Data are presented as adjusted hazard ratios (HRs) for future CVD risk and their 95% confidence intervals, derived from the Fine and Gray’s proportional hazards regression model for the subdistribution of competing risks in which all the variables listed in the table were entered as the explanatory variables, and anti-diabetic, anti-hypertensive, and anti-hyperlipidemic medications were entered as the stratification variables. The explanatory variables except CVD history were centralized to their mean values in the model. CVD, cardiovascular disease; HDL, high-density lipoprotein; LDL, low-density lipoprotein.

## Discussion

The current study, analyzing a clinical database of a prospective observational registry of Japanese patients with diabetes, suggested that the prognostic impact of baseline metabolic profiles on CVD risk would not be identical between patients with diabetes with and without a CVD history.

Risk assessment for future CVD is clinically important, and previous studies have developed risk prediction models for future CVD based on baseline metabolic profiles in patients with diabetes without established CVD [5, 6]. However, a population with diabetes in clinical practice does not comprise solely of those without a CVD history; a CVD history is rather common in real-world settings [20]. Some clinical studies have used the risk prediction models to convert the improvement of metabolic control by an intervention into the estimated absolute risk reduction of future CVD incidence, regardless of patients’ CVD history [8–10]. Others have used the models as a reference to demonstrate that a new biomarker would provide additional information on CVD risk prediction and improve risk stratification in a population including those with a CVD history [21–23]. It is of clinical importance to reveal how different the prognostic impact of respective metabolic profiles would be between patients with a CVD history and those without it.

The current study demonstrated that systolic blood pressure had a significantly different impact on the future CVD risk in patients with a CVD history compared with those without it (*P* for interaction < 0.05). Moreover, the profile was not independently associated with future CVD risk in patients with a CVD history, as in those without it. Previous studies have indicated a beneficial effect of blood pressure control on CVD risk in patients without a CVD history, or in a population wherein most are without a CVD history [24, 25]. Furthermore, previous cohorts confirmed that an elevated blood pressure was a major risk factor for CVD in patients without a CVD history [5, 6]. Hypertension is a well-known accelerator of atherosclerosis and cardiac remodeling, and it would be reasonable that lowering blood pressure reduces future CVD risk in patients without a CVD history. In contrast, the association between blood pressure and CVD risk was controversial in a population with a CVD history, or in one wherein most are with a CVD history. Although some studies showed beneficial effects of lowering blood pressure on CVD risk reduction in patients with a CVD history [26, 27], there is still a clinical concern that lowered blood pressure might reduce perfusion to the brain especially in the presence of arterial stenosis of main vessels [28, 29] and to the distal extremities in patients at risk of CVD [30]. Furthermore, in patients with cardiac dysfunction, a low blood pressure would be a marker of a low cardiac output and be associated with future adverse cardiovascular events [31]. The ACCORD study suggested a tendency for CVD risk reduction by strict blood pressure control [32]. A meta-analysis reported that blood pressure control reduced the risk of stroke, whereas the risk of myocardial infarction was not associated with blood pressure control [33]. It was also suggested that strict blood pressure control would be associated with unfavorable effects on the prognosis [34, 35]. Systolic blood pressure would not be a useful marker for predicting future CVD risk in patients with a CVD history.

Another metabolic profile that had different impacts between patients with and without a CVD history was HDL cholesterol levels. Reduced HDL cholesterol levels were independently associated with future CVD risk in patients without a CVD history, but not in those with it. Reduced HDL cholesterol levels have long been recognized as a classical and familiar risk factor for CVD [36]. However, recent studies adapting the Mendelian randomization method and clinical trials on HDL cholesterol elevation have indicated that HDL cholesterol did not directly modulate CVD risk but rather was just a biomarker [37, 38]. Medications and confounding factors [39] could easily change HDL cholesterol levels. Patients with a CVD history are likely to be on medications; thus, HDL cholesterol levels might not be a useful marker for CVD risk in patients with a CVD history, as in those without a CVD history.

The male sex, an older age, a longer duration of diabetes, higher hemoglobin A1c levels, and higher LDL cholesterol levels were common independent risk factors regardless of a history of CVD. They are well recognized as risk factors for future CVD in a population without a history of CVD [5, 6]. Our findings indicate that they had similar prognostic impact on future CVD risk not only in patients without a history of CVD but also in those with a history. Of the five variables, all except hemoglobin A1c levels were significantly different between patients without a CVD history and those with it. Patients with a CVD history had a higher proportion of male sex, an older age, and longer duration of diabetes, whereas they had lower LDL cholesterol levels. Sex, age, and duration of diabetes can be characterized as the profiles that will not be changed by medical interventions. In a population without a CVD history, male old patients with a long duration of diabetes would be more likely to develop CVD events. It would be reasonable that patients who already developed CVD had a higher proportion of male sex, an older age, and a longer duration of diabetes than those who never developed CVD. On the other hand, hemoglobin A1c and LDL cholesterol levels are clinically modifiable. Lower LDL cholesterol levels in patients with a CVD history suggest that they would receive more intensive intervention to reduce LDL cholesterol levels [40]. On the other hand, hemoglobin A1c levels were not different between the two groups, despite more frequent insulin use in patients with a CVD history, which might reflect more difficulty of glycemic control than of lipid control, as suggested by the Steno-2 study [7].

The CVD incidence rate appeared slightly lower in the current population than in those previously reported in other countries [5, 6, 41, 42]. However, their cohorts enrolled patients decades ago; thereafter, the management of hyperglycemia, hypercholesterolemia, and hypertension drastically improved owing to the accumulation of evidence. These improvements would reduce the overall risk of CVD events. Indeed, our CVD incidence rate was comparable to those in recent cohorts of patients with diabetes in Japan [43, 44]. Ethnic difference might also be another possible reason, because the risk of myocardial infarction is generally lower in the Japanese population than in the Caucasian population, whereas the risk of stroke is not [45]. Such differences might underlie the low CVD incidence in the current study and might have some interaction effects on the associations between metabolic profiles and CVD risk. Future studies in other ethnic populations will be required to validate the current findings.

The current study had some other limitations. First, the registry of 6338 participants was comprised from 464 centers. We did not collect the data regarding how the study subjects were selected from the overall patients attending individual centers. Second, no data were available on family history of CVD, another potential risk factor for future CVD occurrence, or detailed smoking history. In addition, we did not collect detailed information on medication use including dosing and treatment goal achievement, and lifestyle interventions including exercise, rehabilitation, and diets. Different strategies of medication use and lifestyle interventions between patients with and without a CVD history might be a key point potentially confounding and modifying the association of metabolic control with the future CVD risk. Their potential association remained to be revealed. Third, the current study population was limited to Japanese patients aged 40–74 years who were free from proliferative diabetic retinopathy, dialysis-dependent renal failure, and a history of a malignant disease within 5 years. The findings of our study were therefore not generalizable to a wider population, including younger or older age-groups, populations with severe diabetic complications, and other ethnicities. Fourth, no data were available about whether baseline characteristics were different between patients who were eligible but not finally registered in the current study and those registered. Fifth, there would be type II errors due to our insufficient sample size; non-significant associations would not always mean the true absence of the associations. Sixth, we analyzed the association between baseline profiles and future CVD risk. Changes in metabolic profiles during the follow-up period were not considered. Furthermore, the determination of CVD and comorbidities was not based on the codes of the International Classification of Diseases (ICD). CVD events were diagnosed in clinical settings and finally confirmed by the review broad of the working group based on medical records submitted by investigators. The study did not present the diagnostic criteria to investigators in advance or oblige investigators to perform some specific tests for the diagnosis, which would be another study limitation.

In conclusion, the prognostic impact of baseline metabolic profiles on CVD risk would not be identical between patients with diabetes with and without a CVD history, suggesting that it might be inappropriate to apply CVD risk calculators developed for primary prevention to patients with a CVD history.

## Supplementary Information

Below is the link to the electronic supplementary material.

Supplementary file1 (PDF 402 KB)

## Data Availability

The data that support the findings of this study are from the JDCP study group, but restrictions apply to the availability of these data, which was used under license for the current study, and so are not publicly available. Data are however available from the JDCP study group upon reasonable request and with permission of the approval of the relevant ethics committee.
