# Correction to: Different impacts of metabolic profiles on future risk of cardiovascular disease between diabetes with and without established cardiovascular disease: the Japan diabetes complication and its prevention prospective study 7 (JDCP study 7)

**DOI:** 10.1007/s00592-021-01802-x

**Published:** 2021-09-21

**Authors:** Mitsuyoshi Takahara, Naoto Katakami, Yasuaki Hayashino, Rimei Nishimura, Hiroaki Suzuki, Hitoshi Shimano, Narihito Yoshioka, Naoko Tajima, Yoshimitsu Yamasaki

**Affiliations:** 1 Department of Diabetes Care Medicine, Osaka University Graduate School of Medicine, 2-2 Yamadaoka, Suita City, Osaka 565-0871 Japan; 2 Department of Metabolic Medicine, Osaka University Graduate School of Medicine, 2-2 Yamadaoka, Suita City, Osaka 565-0871 Japan; 3 Department of Endocrinology, Tenri Hospital, 200 Mishimacho, Tenri City, Nara 632-8552 Japan; 4 Department of Diabetes, Metabolism and Endocrinology, Jikei University School of Medicine, 3-25-8 Nishi-Shimbashi, Minato-ku, Tokyo, 105-8461 Japan; 5 Department of Endocrinology and Metabolism, Faculty of Medicine, University of Tsukuba, 1-1-1 Tennodai, Tsukuba City, Ibaraki 305-8575 Japan; 6 NTT-East Sapporo Hospital, Minami 1 Jyo Nishi 15 Chome Chuo-Ku, Sapporo City, Hokkaido 060-0061 Japan; 7 Otemachi Place Medical Clinic, 2-3-1, Otemachi, Chiyoda-ku, Tokyo, 100-0004 Japan; 8 Nishi-Umeda Clinic, 3-3-45 Umeda, Kita-ku, Osaka City, Osaka 530-0001 Japan

## Correction to: Acta Diabetologica https://doi.org/10.1007/s00592-021-01773-z

Authors would like to correct the typo in author name of the last author. The author name is corrected from "Yamazaki" to "Yamasaki" in the original publication.

The original article has been corrected.

