# Supplementary material for: Different impacts of metabolic profiles on future risk of cardiovascular disease between diabetes with and without established cardiovascular disease: the Japan diabetes complication and its prevention prospective study 7 (JDCP study 7)
Source: Acta Diabetol. 2021 Aug 30;59(1):57–65. doi: 10.1007/s00592-021-01773-z (PMC8758607; doi:10.1007/s00592-021-01773-z)
Supplement: Supplementary file 1 — Supplementary file1 (PDF 402 KB) [file 592_2021_1773_MOESM1_ESM.pdf]

Study subjects were recruited on an outpatient basis at medical facilities specializing in diabetes, including university hospitals, local base hospitals, and clinics across Japan.

Inclusion criteria:

(i) patients with type 1 and 2 diabetes

(ii) those aged 40 to 74 years.

Exclusion criteria

(i) they were unable to visit their outpatient clinic regularly

(ii) they had proliferative retinopathy

(iii) they were currently on dialysis

(iv) they had been diagnosed with malignancy in the past five years

(v) they were judged by the study investigator to be ineligible for study entry.

↓

7700 eligible patients who gave written informed consent were provisionally enrolled in the JDSP study.

→ 1362 patients who did not meet the study eligibility criteria were excluded.

↓

A total of 6338 patients who met the study eligibility criteria were finally included in the JDCP study.

**Figure S1. Subjects participating in the JDCP study**

## Table S1. Number of observed CVD events

| | Patients without CVD history | Patients with CVD history | Total |
|---|---|---|---|
| Cases who experienced incident CVD during the follow-up period | 307 | 106 | 413 |
| Cases whose first CVD was coronary artery disease | 145 | 53 | 198 |
| Cases whose first CVD was admission for heart failure | 31 | 13 | 44 |
| Cases whose first CVD was cerebrovascular disease | 105 | 32 | 137 |
| Cases whose first CVD was peripheral artery disease | 26 | 8 | 34 |
| Cases who died free from incident CVD | 120 | 29 | 149 |
| Cases who experienced coronary artery disease | 157 | 60 | 217 |
| Cases who experienced admission for heart failure | 35 | 16 | 51 |
| Cases who experienced cerebrovascular disease | 111 | 34 | 145 |
| Cases who experienced peripheral artery disease | 33 | 15 | 48 |

Data are presented as frequency.

## Table S2. Crude CVD incidence (per 1000 person-years)

| | Patients without CVD history | Patients with CVD history | Overall population |
|---|---|---|---|
| CVD (overall) | 9.46 | 32.33 | 11.56 |
| Coronary artery disease | 4.77 | 17.63 | 5.98 |
| Admission for heart failure | 1.05 | 4.46 | 1.38 |
| Cerebrovascular disease | 3.36 | 9.67 | 3.96 |
| Peripheral artery disease | 0.99 | 4.20 | 1.30 |

Data are per 1000 person-years.

## Table S3. Prognostic impact of baseline characteristics on respective components of CVD events

| | Number of observed events<br>Overall (CVD history [-]/[+]) | CVD history [-]<br>Hazard ratio | CVD history [+]<br>Hazard ratio |
|---|---|---|---|
| Male sex | | | |
| Coronary artery disease | 217 (157/60) | 2.67 [1.83, 3.91] | 1.85 [0.96, 3.56] |
| Admission for heart failure | 51 (35/16) | 0.64 [0.33, 1.25] | 0.81 [0.28, 2.34] |
| Cerebrovascular disease | 145 (111/34) | 2.15 [1.40, 3.28] | 1.11 [0.52, 2.39] |
| Peripheral artery disease | 48 (33/15) | 1.74 [0.83, 3.66] | 2.51 [0.57, 11.1] |
| Age (per 10 years) | | | |
| Coronary artery disease | 217 (157/60) | 1.40 [1.14, 1.73] | 0.94 [0.63, 1.39] |
| Admission for heart failure | 51 (35/16) | 2.23 [1.36, 3.67] | 1.26 [0.57, 2.79] |
| Cerebrovascular disease | 145 (111/34) | 1.76 [1.36, 2.29] | 1.28 [0.73, 2.24] |
| Peripheral artery disease | 48 (33/15) | 1.53 [0.96, 2.42] | 3.00 [1.06, 8.54] |
| Type 1 diabetes | | | |
| Coronary artery disease | 217 (157/60) | 0.37 [0.13, 1.03] | 1.10 [0.15, 8.24] |
| Admission for heart failure | 51 (35/16) | - | - |
| Cerebrovascular disease | 145 (111/34) | 0.38 [0.14, 1.07] | 2.01 [0.26, 15.7] |
| Peripheral artery disease | 48 (33/15) | 0.31 [0.04, 2.41] | - |
| Duration of diabetes (per 10 years) | | | |
| Coronary artery disease | 217 (157/60) | 1.41 [1.18, 1.68] | 0.98 [0.72, 1.33] |
| Admission for heart failure | 51 (35/16) | 1.14 [0.76, 1.70] | 1.59 [0.98, 2.57] |
| Cerebrovascular disease | 145 (111/34) | 1.31 [1.06, 1.61] | 1.25 [0.86, 1.82] |

| | | | |
|---|---|---|---|
| Peripheral artery disease | 48 (33/15) | 1.22 [0.82, 1.81] | 1.58 [0.95, 2.62] |
| Smoking history | | | |
| Coronary artery disease | 217 (157/60) | 0.86 [0.62, 1.19] | 1.50 [0.91, 2.49] |
| Admission for heart failure | 51 (35/16) | 1.21 [0.62, 2.37] | 0.65 [0.23, 1.87] |
| Cerebrovascular disease | 145 (111/34) | 1.49 [1.03, 2.16] | 0.72 [0.35, 1.47] |
| Peripheral artery disease | 48 (33/15) | 0.60 [0.28, 1.30] | 1.00 [0.35, 2.80] |
| Body mass index (per 5 kg/m$^2$) | | | |
| Coronary artery disease | 217 (157/60) | 0.99 [0.81, 1.22] | 0.86 [0.58, 1.27] |
| Admission for heart failure | 51 (35/16) | 1.35 [0.92, 1.98] | 0.81 [0.38, 1.75] |
| Cerebrovascular disease | 145 (111/34) | 1.04 [0.82, 1.32] | 0.66 [0.38, 1.14] |
| Peripheral artery disease | 48 (33/15) | 0.74 [0.45, 1.21] | 0.49 [0.21, 1.18] |
| Systolic blood pressure (per 10 mmHg) | | | |
| Coronary artery disease | 217 (157/60) | 1.16 [1.05, 1.28] | 1.01 [0.86, 1.19] |
| Admission for heart failure | 51 (35/16) | 1.28 [1.05, 1.57] | 0.86 [0.62, 1.20] |
| Cerebrovascular disease | 145 (111/34) | 1.14 [1.02, 1.28] | 1.04 [0.83, 1.29] |
| Peripheral artery disease | 48 (33/15) | 1.35 [1.11, 1.65] | 0.95 [0.68, 1.34] |
| HbA1c (per 1% or per 10.9 mmol/mol) | | | |
| Coronary artery disease | 217 (157/60) | 1.16 [1.04, 1.29] | 0.93 [0.73, 1.17] |
| Admission for heart failure | 51 (35/16) | 1.25 [1.05, 1.50] | 1.29 [0.97, 1.71] |
| Cerebrovascular disease | 145 (111/34) | 1.10 [0.96, 1.25] | 1.23 [0.97, 1.57] |
| Peripheral artery disease | 48 (33/15) | 1.10 [0.86, 1.40] | 0.84 [0.50, 1.40] |
| LDL cholesterol (per 20 mg/dl) | | | |

| | | | |
|---|---|---|---|
| Coronary artery disease | 217 (157/60) | 1.16 [1.04, 1.29] | 1.08 [0.91, 1.28] |
| Admission for heart failure | 51 (35/16) | 0.97 [0.77, 1.24] | 1.17 [0.87, 1.59] |
| Cerebrovascular disease | 145 (111/34) | 1.04 [0.91, 1.19] | 0.95 [0.74, 1.21] |
| Peripheral artery disease | 48 (33/15) | 1.01 [0.79, 1.30] | 1.01 [0.70, 1.45] |
| HDL cholesterol (per 10 mg/dl) | | | |
| Coronary artery disease | 217 (157/60) | 0.70 [0.62, 0.79] | 0.96 [0.80, 1.14] |
| Admission for heart failure | 51 (35/16) | 0.77 [0.61, 0.97] | 0.65 [0.43, 0.99] |
| Cerebrovascular disease | 145 (111/34) | 0.70 [0.61, 0.81] | 1.00 [0.80, 1.25] |
| Peripheral artery disease | 48 (33/15) | 0.85 [0.68, 1.07] | 0.90 [0.62, 1.30] |
| Triglycerides (per doubling) | | | |
| Coronary artery disease | 217 (157/60) | 1.28 [1.05, 1.57] | 1.32 [0.95, 1.84] |
| Admission for heart failure | 51 (35/16) | 1.45 [0.95, 2.22] | 1.77 [0.94, 3.31] |
| Cerebrovascular disease | 145 (111/34) | 1.23 [0.95, 1.60] | 0.78 [0.47, 1.30] |
| Peripheral artery disease | 48 (33/15) | 1.34 [0.83, 2.16] | 1.14 [0.46, 2.83] |

Data are presented as hazard ratios (HRs) for future CVD risk and their 95% confidence intervals, derived from the Fine and Gray's proportional hazards regression model for the subdistribution of competing risks in which each variable of interest was entered as the explanatory variable, and anti-diabetic, anti-hypertensive, and anti-hyperlipidemic medications were entered as the stratification variables. Hyphens indicate no estimation due to too small a number of observed events in a subgroup to develop a reliable regression model. CVD, cardiovascular disease; HDL, high-density lipoprotein; LDL, low-density lipoprotein.
